# The Effects of Acorn Origin, Environmental Microbiomes and Local Adaptation on the Leaf Metabolome

**DOI:** 10.1007/s10886-026-01692-9

**Published:** 2026-02-13

**Authors:** Chandrasekar Ramanathan, Lisse Goris, Arti Mishra, Jenna Lihavainen-Bag, Katharina Pawlowski, Benedicte Riber Albrectsen, Ayco J.M. Tack

**Affiliations:** 1 Department of Ecology, Environment and Plant Sciences, Stockholm University, Stockholm, SE-106 91 Sweden; 2 Department of Plant Science, Umeå University, Umeå Plant Science Centre, Umeå, SE-907 36 Sweden; 3 Department of Botany, Hansraj College, Delhi University, Delhi, India

**Keywords:** GC-MS, Local adaptation, Metabolomics, Microbiome, Plant-microbe interactions, Quercus robur

## Abstract

Plants are associated with microbial communities, which are inherited through the seed and acquired from the environment. These microbiomes influence plant physiology, chemistry, and functioning. Yet, we lack insights into how seed origin and the environmental microbiome jointly influence the leaf metabolome. We used untargeted metabolomics (gas chromatography/mass spectrometry) on leaves of pedunculate oak (*Quercus robur*) seedlings to examine metabolic responses to different seed origins and environmental microbiomes, as well as home and away environments. For this, acorns were collected from three mother trees and grown in a multifactorial design with soil and canopy microbiomes originating from the local mother tree (i.e., the home treatment) and neighbouring trees (i.e., the away treatment). We also measured two plant traits—plant height and leaf chlorophyll content—to examine relationships between plant traits and the metabolome. The leaf metabolome did not differ significantly between plants growing with different soil and canopy microbiomes. However, the leaf metabolome differed among acorn origins and between seedlings growing in home vs. away treatments. We found no clear link between plant traits and the leaf metabolome. This study is one of the first to disentangle the combined effects of seed origin and environmental microbiomes on plant leaf chemistry, and the home vs. away framework provides novel insights into local adaptation effects on plant metabolomes within forest ecosystems. These findings have practical implications for the use of local genotypes and the development of microorganism-based management practices in sustainable forestry and agriculture.

**Supplementary Information:**

The online version contains supplementary material available at 10.1007/s10886-026-01692-9.

## Introduction

The interaction between a plant and its microbiome, and their joint genetic expression, is well-known to drive several aspects of plant functioning, including nutrient uptake, stress tolerance and defence against pathogens and insects (Matsumoto et al. 2021). Interactions between a plant and its microbiome are also expected to cause shifts in plant chemistry, and shifts in plant chemistry affect the composition of the plant microbiome (Pang et al. 2021). For example, Huberty et al. (2020a, b) used genetically identical plants to demonstrate that soil microbiomes differ in the metabolic responses they elicit, while Kudjordjie et al. (2021) showed that chemically distinct *Arabidopsis* mutants recruit different microbiomes from the same soil microbiome source. Yet, most studies evaluating the effect of microbiomes on plants have focused on plant fitness and functional traits, with metabolomes receiving little attention (Yergeau et al. 2015; Rodríguez et al. 2020). Unravelling the effect of microbiomes on metabolomes can improve our mechanistic understanding of how plant-microbiome interactions influence plant functioning (Walker et al. 2022). Such insights are important for our understanding of ecosystem functioning in nature (Rodrigues et al. 2021), as well as the development of sustainable, microorganism-based management practices in forestry and agriculture.

**Fig. 1:**
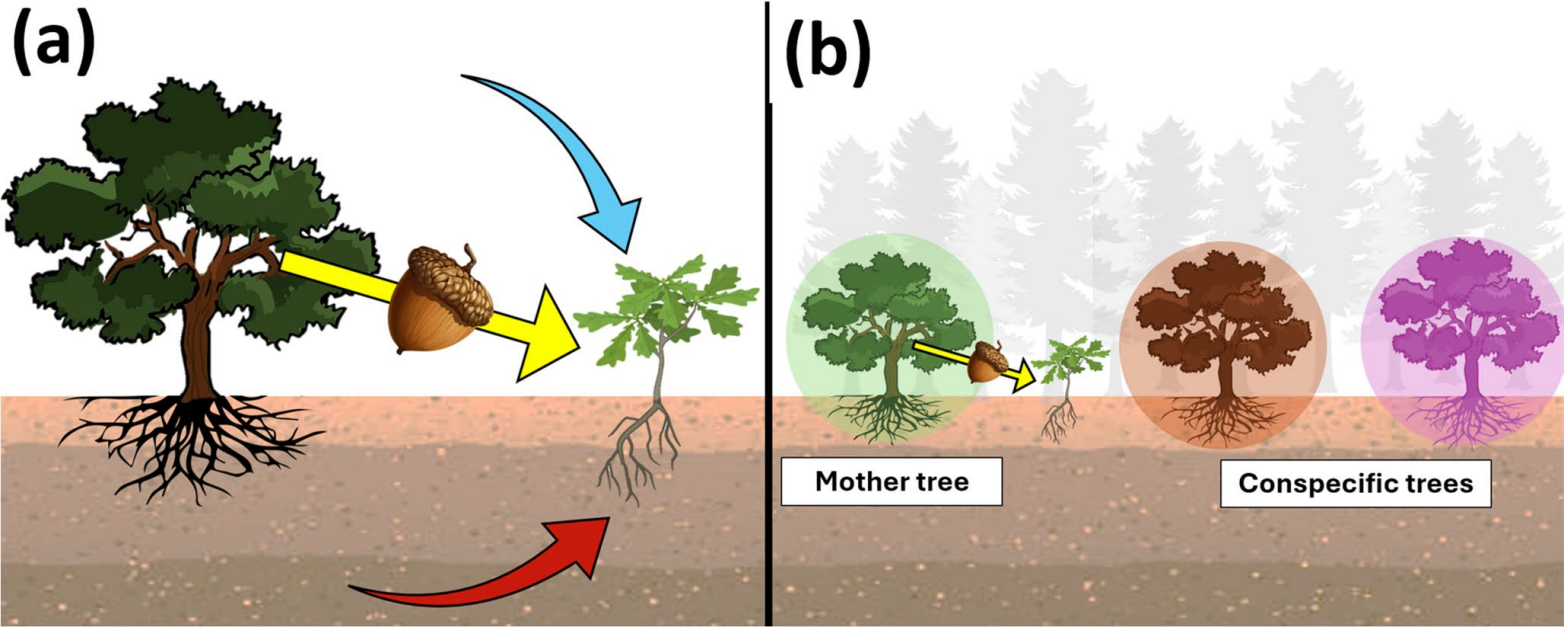
Conceptual overview of the study. Figure 1a illustrates the vertical (i.e., inherited) and horizontal (i.e., environmental) sources of microbial communities for a seedling. The yellow arrow represents the vertical transmission that occurs through the seed, the red arrow represents horizontal transmission through the soil, and the blue arrow represents horizontal transmission through the air. Figure 1b illustrates an instance of local adaptation at the individual tree level. In this illustration, the home tree (green) is an acorn source and thus represents the home environmental microbiome for a seedling growing from its acorn, and the other trees (brown and lilac) are neighbouring conspecific trees that represent away environmental microbiomes for a seedling from the green mother tree

A developing plant acquires its microbiome vertically and horizontally. The vertically transmitted community consists of those microorganisms that are passed down from parent to offspring. In seed plants, this occurs through the seed (yellow arrow in Fig. 1a). Previous studies have focused on how either seed microbiomes or environmental microbiomes affect the metabolome of seedlings. Due to difficulties involved in culturing plants without seed microorganisms, our understanding of the function of the vertically transmitted microbiome remains limited (Chen et al. 2018). Nevertheless, several studies have shown that variation in seed microbiomes is a major driver of differences between plant metabolomes (Deepak et al. 2018; Hayashi et al. 2023). Horizontal transmission refers to microorganisms acquired from environmental microbiome sources such as soil and plant canopies (blue and red arrows in Fig. 1a). Adult plants play a central role in regulating the environmental microbiome sources of seedlings in natural ecosystems, as they drive soil processes and cover the seedlings with their canopies (Womack et al. 2015; Eck et al. 2019; Hannula et al. 2021). A major outstanding question is then how the different adult plant-driven vertical and horizontal microbiome sources jointly shape the metabolome of the developing seedling. Many studies have pointed out that individual plants modify the soil microbiome surrounding their root system, which in turn affects the growth, physiology and metabolomes of subsequent generations of plants (Huberty et al. 2022). On the other hand, the canopy microbiome—i.e., the phyllosphere microbiome associated with adult trees—and its role as a microbiome source for seedlings growing underneath the adult trees has received comparatively little focus. While canopy-to-seedling transfer of microorganisms might occur extensively in forest ecosystems (Womack et al. 2015), no study thus far has looked at canopy microbiome effects on seedling performance and metabolomes.

While traditional research on local adaptation has largely focused on abiotic factors like heavy metals and soil nutrients (Briscoe Runquist et al. 2020), recent studies using reciprocal transplant experiments of plants from different populations have shown that local microbiomes can lead to microorganism-mediated local adaptation or maladaptation (Petipas et al. 2021; Brady and Farrer 2024). However, heterogeneity in environmental microbiomes exists even within plant populations, where plant microbiomes will differ not only between individuals belonging to different plant species, but also between individuals belonging to the same plant species (Albrectsen et al. 2018; Habiyaremye et al. 2021; Malacrinò et al. 2022; Khasanova et al. 2023). As plants have coevolved with their microbiome (Abdelfattah et al. 2022; Addison et al. 2025), this raises the possibility that seedlings are adapted to the local microbiome of their parents (Fig. 1b). Yet, there exists little research on microbiome-mediated local adaptation, especially at small spatial scales (Addison et al. 2024). As adult tree microbiomes have been shown to affect seedling survival, performance and responses to biotic stress (Vivas et al. 2013, 2017), microbiome-mediated local adaptation is expected to be also reflected in the seedling metabolome. However, to the best of our knowledge, local adaptation effects on the plant metabolome have not yet been studied. Investigating local adaptation effects on plant chemistry is important for developing microorganism-based forestry management strategies, such as the use of tree genotypes adapted to the local microbiome (Jones 2013; Boshier et al. 2015; Dupré la Tour et al. 2020), or the inoculation of seedlings with their local microbiomes (Rúa et al. 2016; Beattie et al. 2024; Gomes et al. 2025).

In this study, we explored how seed origin, soil microbiomes, and canopy microbiomes jointly affect the leaf metabolome of pedunculate oak seedlings. The pedunculate oak is a keystone species that supports a rich biodiversity and is one of the most common species of oak in Europe (Ernst, 1965). We examined these effects at a small spatial scale by using microbiomes and acorns stemming from adult trees growing within a single population. Acorns were collected from three trees and were grown with soil and canopy microbiomes originating from their mother tree (i.e., home treatment) and neighbouring trees (i.e., away treatment). We utilized the home and away treatments to explore patterns of local adaptation. In this study, we use the term *local adaptation* to refer to metabolic differences between home and away environments that are consistent with local adaptation patterns, but we do not directly measure adaptation in terms of fitness differences between the plants. We used untargeted gas chromatography/mass spectrometry (GC-MS) to analyse the metabolome. We also measured two plant traits—plant height and leaf chlorophyll content—to complement the metabolomics. More specifically, we focused on three questions:

1. What is the impact of the soil microbiome, canopy microbiome, acorn origin, and home vs. away treatments on the leaf metabolome?
2. What is the impact of the soil microbiome, canopy microbiome, acorn origin, and home vs. away treatments on plant height and leaf chlorophyll content?

## Methods and Materials

### Collection of Acorns, Soils and Canopies

Acorns were collected from three trees on Stockholm University campus (59°21’58.5"N 18°03’43.0"E) located in the Royal National City Park in Norra Djurgården. To test for small-scale local adaptation, we selected three trees within the same population that were located c. 10 to 15 m apart. Acorns from the three trees were stored in a 4 °C cold room during winter. In spring, the acorns were rinsed with water and germinated on cotton in a controlled climate chamber. After two weeks, acorns were transferred to the treatment pots. To obtain the soil microbiome associated with each of the mother trees, we collected soil from beneath the canopy of each mother tree. To obtain the canopy microbiome associated with each of the mother trees, we regularly collected twigs with leaves from each of the mother trees using a sterile pruning shear. Before using the twigs, we removed infested leaves to reduce the risk of insect infestation.

### Experimental Setup and Sampling

**Fig. 2:**
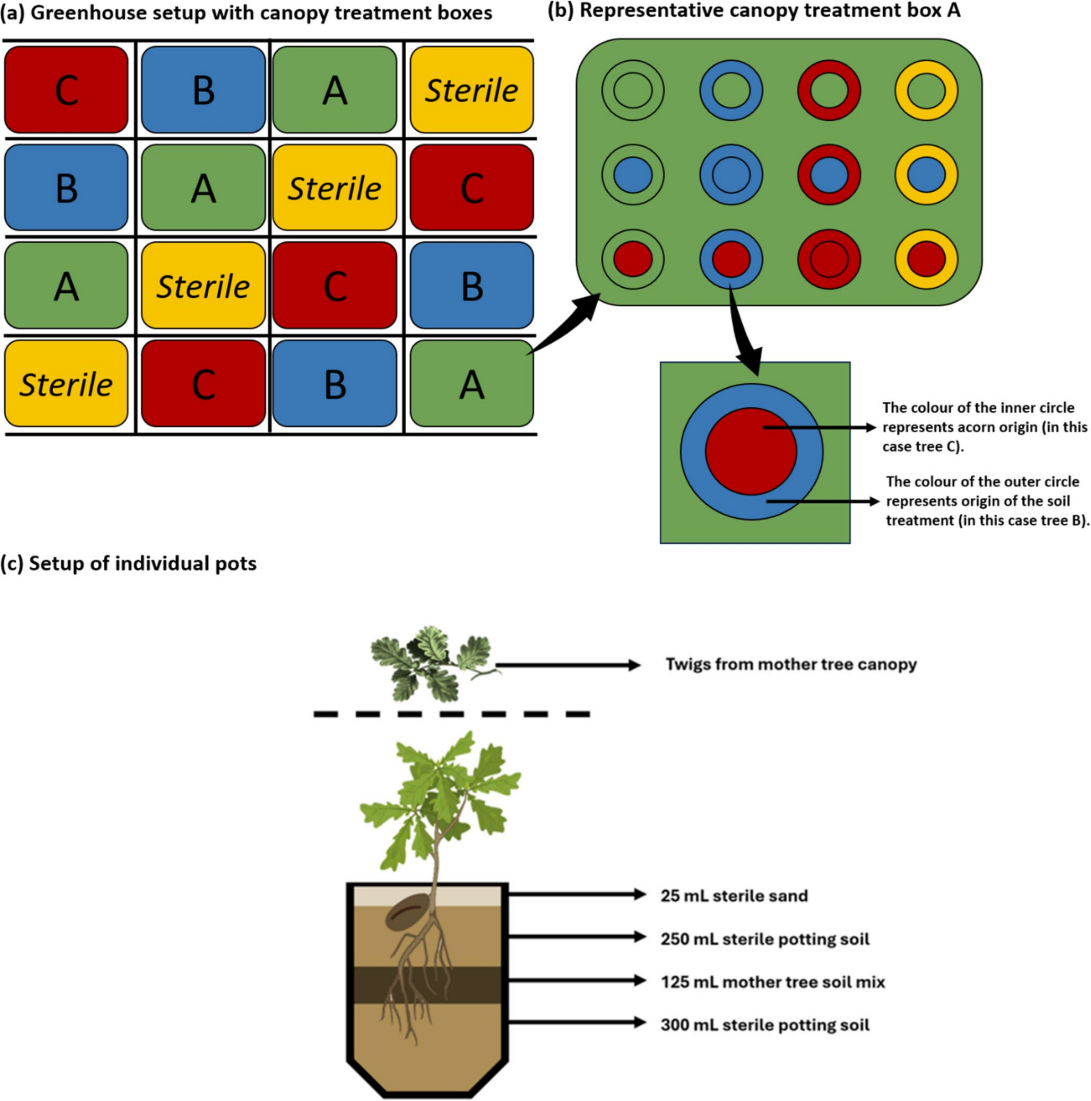
Graphical overview of the hierarchical setup of the experimental design. Panel (**a**) shows the spatial layout of the greenhouse setup at the canopy treatment level, with different canopy treatments shown in the figure as differently coloured boxes (red for the canopy microbiome of mother tree A, green for B, blue for C and yellow for the sterile control where no twigs are used). Panel (**b**) shows an example of a canopy treatment box belonging to the mother tree A. Within each canopy treatment box, twelve seedlings were placed, as represented by circles. The colour of the outer circle represents the soil treatment while the inner circle represents acorn origin. Panel (**c**) illustrates the setup of the individual plant pots. The soil treatment for each acorn was prepared by combining 550 mL potting soil, 25 mL sand and a 125 mL soil mix made up of equal parts A, B and C soil with only the designated treatment soil added unsterilized. For the sterile soil treatment, the soil mix was made with equal parts of sterilized A, B and **C** soil types. The canopy treatment consisted of twigs from the respective mother tree placed on a mesh wire above the seedlings

To disentangle the relative effects of acorn origin, soil microbiome and canopy microbiome on the leaf metabolome, we set up a multifactorial greenhouse experiment using germinated acorns (Fig. 2). For this experiment, we combined three different acorn origins (A, B and C) with four soil microbiome treatments (A, B, C and a control treatment) and four canopy microbiome treatments (A, B, C and a control treatment). Every treatment combination had 4 replicates, leading to a total of 192 plants (Fig. 2ab). The conditions in the greenhouse were 20 °C during the day (10 h) and 10 °C during the night (14 h). The relative humidity was maintained at 60%.

To establish the soil microbiome treatments, we planted the germinated acorns individually into pots that contained one of four soil treatments: (i) the soil microbiome associated with tree A, (ii) the soil microbiome associated with tree B, (iii) the soil microbiome associated with tree C, and (iv) a sterile soil treatment (i.e., the control). The pot with soil for each treatment was prepared by combining 550 mL sterile potting soil, 25 mL sterile sand and a 125 mL soil mix (Fig. 2c). The 125 mL soil mix for treatments A, B and C was made up of equal parts of A, B and C soil with only the designated treatment soil left unsterilized. Soil was sterilized in batches of c. 5 L under a sterilization temperature of 121 °C for 30 min. While this procedure may have affected soil abiotic properties, we used it as an effective method to biotically clear soil (King et al. 2024). In addition, we used a large proportion of sterile background potting soil (550 mL potting soil to 125 mL soil mix) to minimize differences in abiotic soil characteristics between treatments that might result from sterilizing different parts of the soil mix for different treatments (van Dijk, Abdelfattah et al. 2022). For the sterile soil control, the soil mix was made with equal parts of sterilized A, B and C soil types.

To establish the canopy microbiome treatments, we tried to simulate natural conditions and processes by placing twigs from the three mother plants above the plant pots, creating four canopy treatments: (i) the canopy microbiome from tree A, (ii) the canopy microbiome from tree B, (iii) the canopy microbiome from tree C, and (iv) no canopy microbiome (i.e., the control, where no twigs were used). To allow microorganisms to disperse with gravity, twigs were placed atop a horizontal fence of chicken wire, under which the seedlings from a given block were placed (Fig. 2a). To simulate natural dispersal by rain, we watered from the top using a watering nozzle, so water would go through the twigs. To avoid drying of the twigs, they were placed in 50 mL Falcon tubes filled with water. The twigs were checked twice a week and replaced when they showed signs of wilting. The advantage of this novel approach of utilizing twigs to simulate the natural canopy-to-seedling transfer of microbiomes is that it allows for experimental manipulation within the controlled setting of a greenhouse, along with any other set of experimental manipulations (in this case, the soil microbiome). At the same time, it is important to note that the naturalness of the canopy treatment is also limited by the controlled conditions in the greenhouse, as there are less fluctuations in temperature, and no wind and insects to disperse spores.

**Fig. 3:**
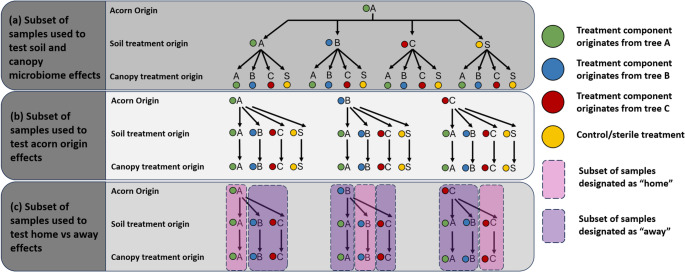
The subsets of plants used for looking at the effects of (**a**) soil and canopy microbiome, (**b**) acorn origin, and (**c**) home vs. away environments. Each treatment combination has four replicates. In total, 96 plants were analyzed

To investigate the effect of soil and canopy microbiome treatments, we used seedlings from a single acorn origin (i.e., acorn origin A; Fig. 3a). To investigate the effect of acorn origin, we used seedlings from all three acorn origins growing in home environments (where the acorn origin, the soil treatment, and the canopy treatment were all from the same tree), away environments (where the acorn origin differed from the soil and canopy treatments), and in sterile environments (where the acorn was growing in both the control soil treatment and control canopy treatment; Fig. 3b). It is important to note that while we investigated the effect of acorn origin, we did not attempt to disentangle whether this acorn origin effect is due to the vertically transmitted acorn microbiome or due to genetic factors. To test for local adaptation effects, we used the subset of seedlings growing in home environmental microbiomes (i.e., environments with both home soil and home canopy treatments) and away environmental microbiomes (i.e., environments with both away soil and away canopy treatments; Fig. 3c). In total, we analysed 96 out of the 192 plants included in the full factorial design (Fig. 3).

### Trait Measurements

Seven weeks after potting (i.e., nine weeks after germination), we measured the height of each sampled seedling and recorded the chlorophyll content index (cci) of one of the top leaves using a Opti-Sciences CCM-200 plus Chlorophyll Content Meter. We selected plant height as it is considered the most important indicator of plant fitness during the seedling stage of forest species (Grossnickle 2012; Gardiner et al. 2019) and leaf chlorophyll content for its relationship with leaf metabolism (Croft et al. 2017).

### Metabolomic Analyses

Seven weeks after potting, we collected one of the top leaves from the first flush for metabolomic analysis. This time period is sufficient for oak seedlings to establish roots that interact with the soil microbiome treatments, as demonstrated by previous studies that detected clear differences in seedling microbiome composition, as well as metabolic responses of the seedling, after similar time frames (van Dijk, Abdelfattah et al. 2022; Van Dijk et al. 2022). For this, the leaf was cut using sterile scissors, after which the midrib was excised and the leaf snap-frozen in liquid nitrogen before storage in − 80 °C. The frozen leaf samples were ground in liquid nitrogen using a mortar and a pestle and a subsample of ~ 10 mg was used for metabolomic analysis For extraction, 1000 µL of extraction mixture (1/1/3 v/v/v chloroform/water/methanol) including internal standards (2.5 ng µL^− 1^ L-proline-13C5, 2.5 ng µL^− 1^ alpha-ketoglutarate-13C4, 2.5 ng µL^− 1^ myristic acid-13C3, 7.5 ng µL^− 1^ cholesterol-D7, 7.5 ng µL^− 1^ succinic acid-D4, 0.0625 ng µL^− 1^ salicylic acid-D6, 2.5 ng µL^− 1^ L-glutamic acid-13C5,15 N, 7.5 ng µL^− 1^ putrescine-D4, 7.5 ng µL^− 1^ hexadecanoic acid-13C4, 2.5 ng µL^− 1^ D-glucose-13C6 and 2.5 ng µL^− 1^ D-sucrose-13C12) was added to each sample along with two tungsten beads (3 mm diameter, Qiagen). The samples were homogenized in a Retsch GmHB MM400 mixer mill for 3 min at 30 Hz. The samples were then centrifuged at 4 °C at 14,000 rpm for 10 min and supernatants were transferred into new vials. An aliquot of 200 µL was transferred into an insert in a LC vial and dried in a vacuum centrifuge (SpeedVac). Sample blanks without plant material were included in the analysis to account for background signals. Quality control (QC) samples were prepared by combining aliquots of sample extracts. Dried samples were stored at − 80 °C prior to GC/MS analysis.

Derivatization was performed according to Schauer et al. (2005). Samples were thawed and dried in a vacuum for 5 min before derivatization with 30 µL of methoxyamine (15 µg µL^− 1^ in pyridine). The sample vials were then capped, vortexed for 10 min and incubated overnight (16 h) at room temperature. The samples were then derivatized with 30 µL of MSTFA containing 1% TMCS, and 30 µL heptane including 15 ng µL^− 1^ of methyl stearate was added to each sample. The samples were vortexed and incubated at room temperature for 1 h before GC/MS analysis. The details of GC/MS analysis and data pre-processing are provided in Text S1.

### Statistical Analyses

From the raw GC/MS data, 52 metabolites were annotated. Five samples that failed quality control were removed before analysis. Data was first normalized with multiple internal standards using Cross-Contribution compensating Multiple standard Normalization (CCMN), which is based on a principal component analysis (PCA) model (Redestig et al. 2009). Subsequently, data were normalized based on the fresh weight of the sample. Multivariate statistical analyses were conducted with the normalized data using SIMCA v18.0.0 (Umetrics, Umeå, Sweden).

We visualized differences in the metabolic profiles between soil microbiome treatments, canopy microbiome treatments, acorn origins and home vs. away environments using a principal component analysis (PCA). For the PCA and OPLS-DA, we applied log transformation to the data to reduce skewness (Li et al. 2016). If visual separation was validated in the PCA, we conducted a supervised Orthogonal Projections to Latent Structures Discriminant Analysis (OPLS-DA). To obtain “metabolites of special interest” for univariate analyses, we utilized the VIP scores (Variable Importance in Projections) from the OPLS-DA models and selected metabolites with VIP ≥ 1. These metabolites are shown in Tables S2 and S3.

We further modelled the concentration of each metabolite as a function of soil, canopy, acorn and home/away environments with one-way ANOVAs using the function *lm* in base R v4.3.3 (R Core Team, 2024). If the ANOVA showed a significant treatment effect (*p* < 0.05), we conducted *post hoc* pairwise comparisons with Tukey’s HSD to control for the family-wise error rate. In cases where the residuals were not normally distributed or had unequal variances, we used Kruskal-Wallis tests as followed by post-hoc Dunn tests with Holm-Bonferroni adjustment using the function *dunnTest* in package *FSA* (Ogle et al. 2020). We used similar models to analyse the two measured plant traits—seedling height and leaf chlorophyll content.

## Results

### Impact of the Soil Microbiome, Canopy Microbiome, Acorn Origin and Home vs. Away Treatments on the Leaf Metabolome

**Fig. 4:**
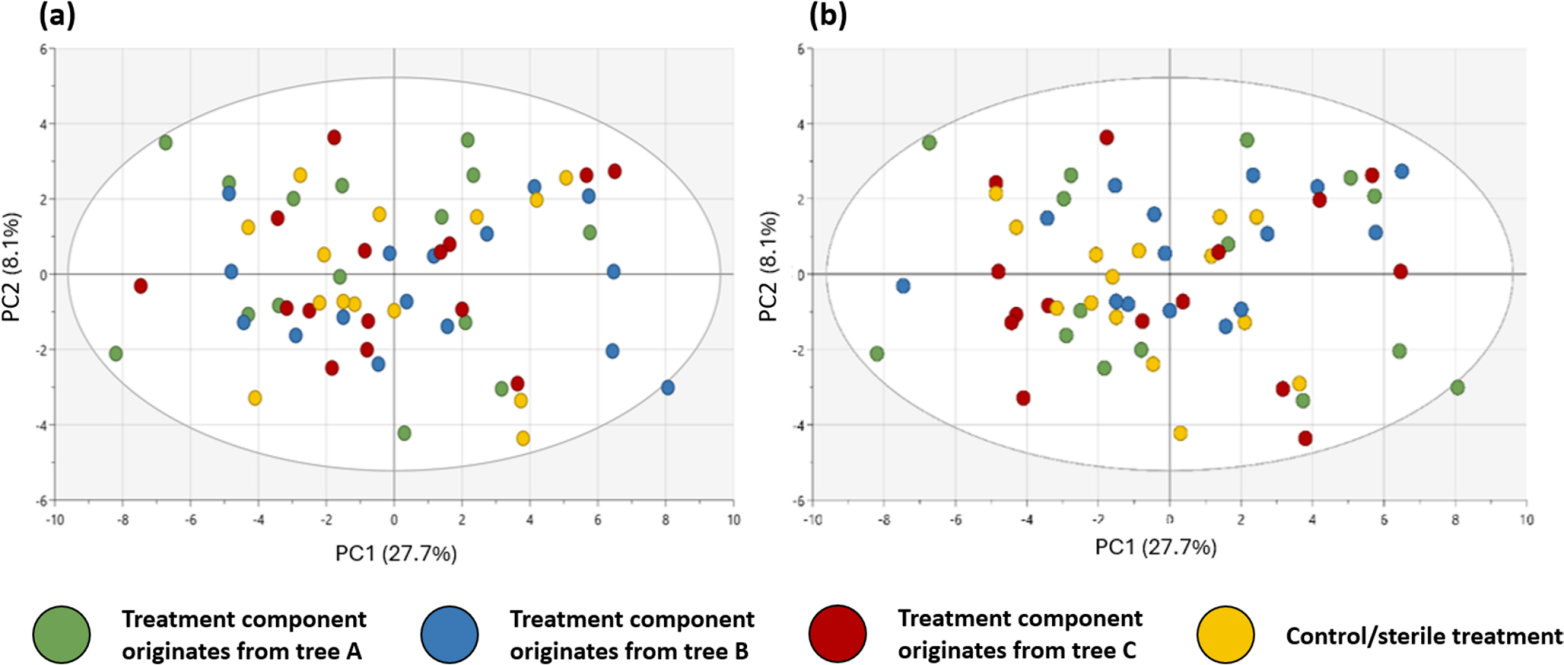
The leaf metabolic profile of oak seedlings in response to different (**a**) soil microbiome and (**b**) canopy microbiome treatments, as visualized in PCA (Principal component analysis) score plots. The colours in panel (**a**) represent the origin of the soil microbiome (soil A, soil B, soil C and sterile soil) and the colours in panel (**b**) represent the origin of the canopy microbiome (canopy A, canopy B, canopy C, and control). Both PCA models were autofit to two components with R2X(cum) of 0.358 and Q2(cum) of 0.208

The leaf metabolic profile did not differ between the soil microbiome treatments, or between the canopy microbiome treatments (Fig. 4). While there was no overall treatment effect, a few of the 52 metabolites still differed significantly in univariate analyses: allothreonine and serine differed between soil microbiome treatments and ellagic acid, 1,5-anyhdrosorbitol, alpha-tocopherol, cycloartenol, delta-tocopherol, gamma-tocopherol, and glucuronic acid differed between canopy microbiome treatments (Table S1).

**Fig. 5:**
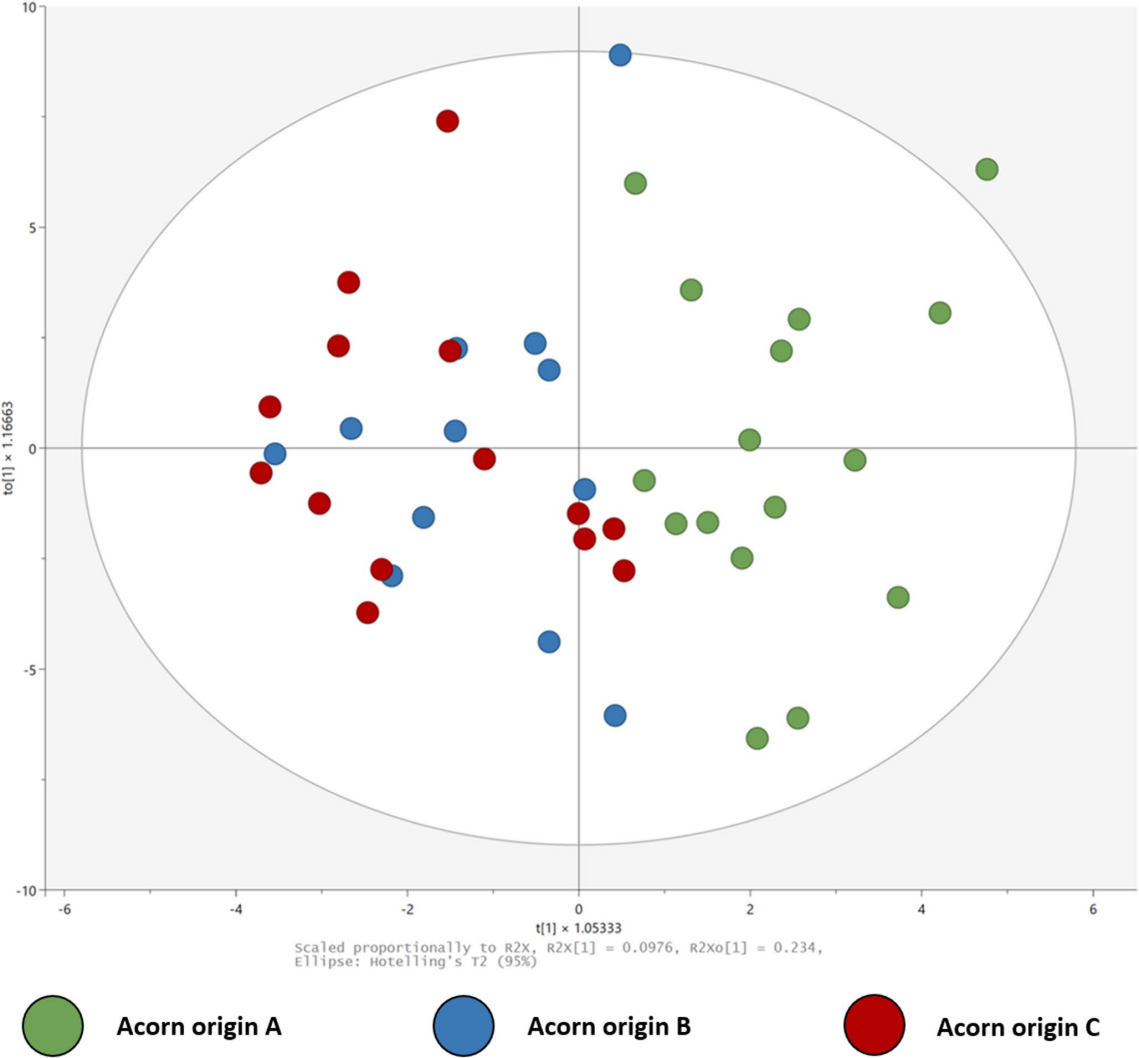
The leaf metabolic profile of oak seedlings in response to acorn origins, as visualized in PCA (Principal component analysis) score plots. The colours represent different acorn origins. The PCA model was autofit to five components with R2X(cum) of 0.597 and Q2(cum) of 0.208. For the visualization of the OPLS-DA model between acorn origins, see Fig. S1

The leaf metabolic profile differed between acorn origins, with A separating from B and C along the second component (Figs. 5 and S2). Eight individual metabolites were found to significantly differ between acorn origins in univariate analyses: citric acid, delta-tocopherol, dehydroascorbic acid, glucose, glucuronic acid, kaempferol, succinic acid and N-methyl trans-4-hydroxy-L-proline (2 S,4R)−4-hydroxy-1-methyl pyrrolidine-2-carboxylic acid (UHMOGBXQYJLCHX-IMSYWVGJSA-N) (Table S2).

**Fig. 6:**
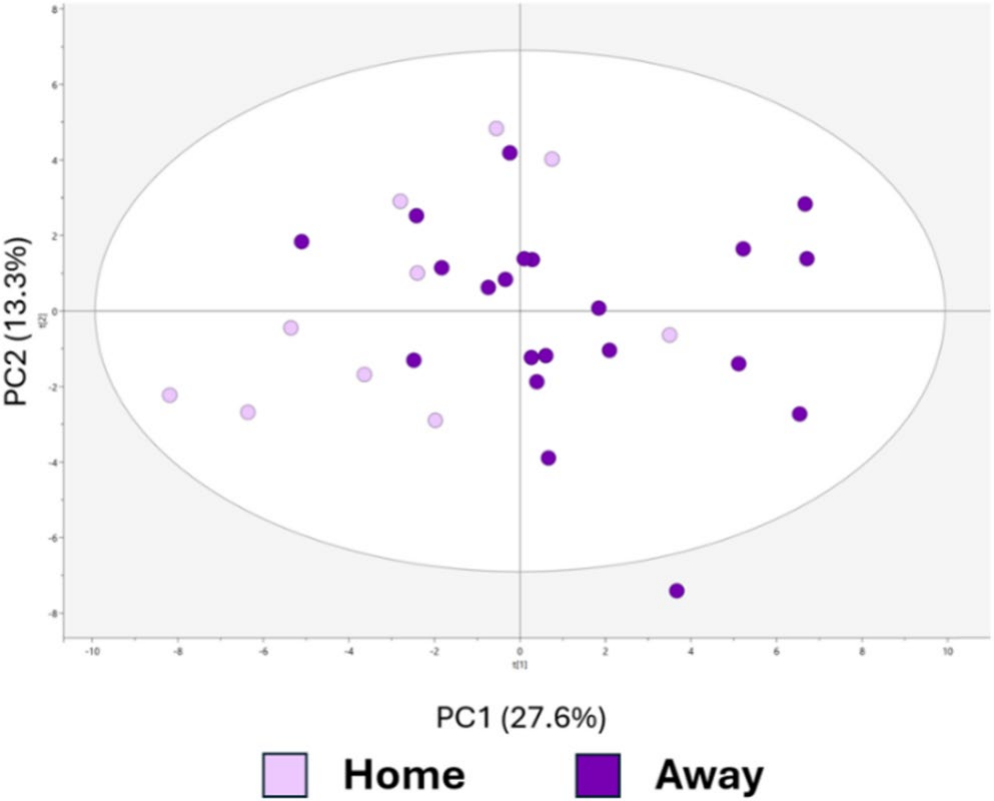
The leaf metabolic profile of oak seedlings grown in home and away environments, as visualized in PCA (Principal component analysis) score plots. The colours represent the home and away environments. The PCA model was autofit to three components with R2X(cum) = 0.496 and Q2(cum) = 0.208. For the visualization of the OPLS-DA model between home and away environments, see Fig S2

The leaf metabolic profile differed between the home and away treatments (Figs. 6 and S3). Fifteen individual metabolites differed significantly between the home and away treatments in univariate analyses: alanine, allothreonine, alpha-linolenic acid, beta-sitosterol, ethanolamine, glutamic acid, glyceric acid, glycine, malic acid, palmitic acid, quinic acid, scyllo-inositol, serine, sucrose and threonine (Table S3). All metabolites except malic acid were significantly higher when seedlings were grown in away environments compared to seedlings grown in home environments.

### Impact of the Soil Microbiome, Canopy Microbiome and Acorn Origin on Plant Traits

**Fig. 7:**
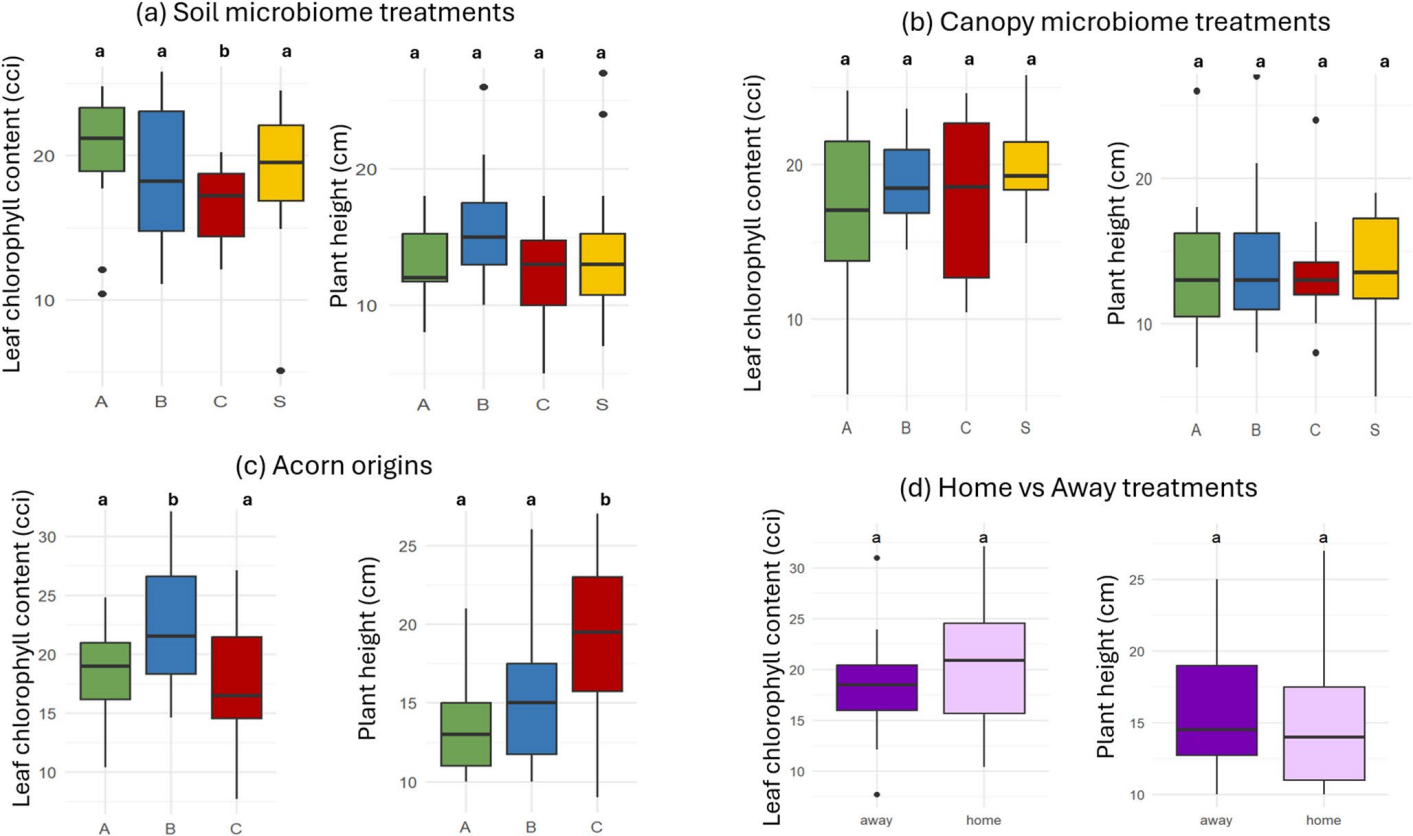
The effect of soil and canopy microbiome, acorn origin and home vs. away treatments on the plant traits plant height and leaf chlorophyll content. Shown are box plots, separately for the (**a**) soil microbiome, (**b**) canopy microbiome, (**c**) acorn origins, and (d) home vs. away treatments. Significant differences between treatment are noted by a letter above the boxes, where groups with the same letter do not differ

Chlorophyll content, but not plant height, differed significantly between soil microbiome treatments (Fig. 7). However, the only pairwise difference in chlorophyll content was between soil treatment C and the sterile treatment (Table S5). Plant height and chlorophyll content did not differ significantly between the canopy microbiome treatments. Both plant height and chlorophyll content differed significantly between acorn origins (Table S4). Chlorophyll content differed significantly between the A & B and B & C acorn origin pairs, and plant height differed significantly between the A & C and B & C pairs (Table S5). Neither trait differed significantly between the home and away treatments (Fig. 7).

## Discussion

Our study investigated how environmental microbiomes and seed origin affect the oak seedling leaf metabolome and patterns of local adaptation. While seed origin had a strong effect on the metabolome, we did not detect a direct imprint of the soil and canopy microbiome on the leaf metabolome. However, seedlings growing in home environments differed in their metabolome from seedlings growing in away environments. The role of the environmental microbiome and seed origin in shaping the leaf metabolome and local adaptation patterns improves our understanding of the processes underlying plant-microbe interactions in natural ecosystems and might inform the use of local tree genotypes and the application of microbiomes in sustainable forestry (Wei and Tan 2023).

We did not detect a direct imprint of the soil microbiome on the leaf metabolome. While our findings contrast with a recent study demonstrating that plant-induced changes in soil microbiomes alter the metabolomes of plants that later grow in these soils (Huberty et al. 2020), they corroborate a previous study demonstrating a weak effect of the soil microbiome on the leaf metabolome of pedunculate oak seedlings (Van Dijk et al. 2022a; Dijk et al. 2022b, c). Likewise, we found that canopy environmental microbiomes did not affect the seedling leaf metabolome. As no other studies have looked at the effects of the microbiome originating from the canopy on the leaf metabolome of seedlings, the lack of a canopy effect on the seedling metabolome is difficult to interpret. One possible explanation for the lack of independent soil and canopy effects on the leaf metabolome could be the time of sampling in our study. Some studies indicate that in the early stages of seedling development, seed-transmitted microorganisms dominate the seedling microbiome and are the primary drivers of plant chemistry (Johnston-Monje and Raizada 2011; Abdelfattah et al. 2023), and only as a seedling matures, it develops increased interactions with environmental microorganisms (Latz et al. 2015). Studies conducted with poplar trees (*Populus* spp.) found that the root and shoot microbiome of seedlings changed drastically during the first months of growth (Dove et al. 2021), and that this shift in the microbiome triggered substantial changes in the seedling metabolome (Mangeot-Peter et al. 2020; Xie et al. 2023). While species-specific differences between oaks and poplars could exist, we hypothesize that oak seedlings grown for a longer time would show increasing effects of the environmental microbiomes on the metabolome. Another explanation for the lack of independent soil and canopy effects on the leaf metabolome could be that the soil and canopy microbiomes were only weakly differentiated among our three trees; however, research on within-population variation in tree microbiomes is limited and shows that the amount of variation depends on tree species and environmental contexts (Saetre and Bååth 2000; Nacke et al. 2016; Craig et al. 2024). Finally, we tested the independent soil and canopy effects on the leaf metabolome for a single acorn origin, and we hope that future studies will confirm (or refute) our findings by comparing origins from multiple trees, populations and regions.

In contrast to the absent or weak effects of the soil and canopy microbiomes, we found a strong independent effect of acorn origin on the leaf metabolome. While we note that we cannot pinpoint whether the effect of acorn origin is driven by the microbiome transferred from the mother tree, plant genetics, or a combination of both, our result aligns with the established theories about the dominance of seed microorganisms at early stages of seedling development and that differences in seed microbiomes drive diversity in plant metabolomes (Müller et al. 2020; Hayashi et al. 2023). Such differences in metabolomes are known to exist between plant individuals belonging to the same population, and this within-population metabolic heterogeneity is hypothesized to deter herbivore and pathogen outbreaks (Züst et al. 2012; Moore et al. 2014; Müller et al. 2020). As a future direction, it would be interesting to test the effect sizes of among-tree genetic differentiation vs. the inherited microbiome in driving these patterns.

Two metabolites, serine and allothreonine, significantly differed between soil microbiome treatments. The amino acid serine is a precursor of allothreonine, which is a non-proteinogenic amino acid. Both serine and allothreonine have been linked to stress responses in plants (Kefale et al. 2024). The metabolites that differed between canopy treatments were all secondary metabolites: tocopherols, which are associated with chloroplasts and redox homeostasis in leaves (Kanwischer et al. 2005), the phenolic ellagic acid, the steroid precursor cycloartenol, glucuronic acid, which is a hemicellulose precursor (Ishihara et al. 2017; Tietel et al. 2020) and 1,5-anhydrosorbitol. The metabolites that differed between acorn origins were citric acid, delta-tocopherol, dehydroascorbic acid, glucose, glucuronic acid, kaempferol, succinic acid and N-methyl trans-4-hydroxy-L-proline (2 S,4R)−4-hydroxy-1-methyl pyrrolidine-2-carboxylic acid (UHMOGBXQYJLCHX-IMSYWVGJSA-N). Delta-tocopherol, which also differed between canopy treatments, has been extensively described in the literature as integral to plant defence (Wedow et al. 2021), and is linked to dehydroascorbic acid (DHAA) levels (Kanwischer et al. 2005). Glucose was the sole sugar that differed significantly. Glucose levels modulate several physiological processes in plants (Siddiqui et al. 2020). Kaempferol is a flavonoid and a known antioxidant in plants (Kumari et al. 2024). Among the acids that differed, glucuronic acid is a hemicellulose precursor (Tietel et al. 2020), and citric acid and succinic acid are TCA cycle intermediates (Choi et al. 2021).

The leaf metabolic profile differed between the home and away environments, indicating an interactive effect between acorn origin and the environmental microbiomes. Several studies focusing on plant performance and fitness traits have demonstrated microbiome-mediated local adaptation between plant populations using reciprocal transplant experiments (Johnson et al. 2022; Kazarina et al. 2023; Khasanova et al. 2023). With regards to tree species, a single study utilizing pine found that seedlings grown in home environments with the local soil microbiome performed better when facing climatic disturbances when compared to seedlings grown in away environments (Remke et al. 2020). While soil microbiome-mediated local adaptation has been studied extensively in terms of plant performance and fitness, the role of tree canopy microbiomes in local adaptation remains unexplored. Likewise, no other studies have addressed the impact of microbiome-mediated local adaptation on the seedling metabolome. Our study indicates that local adaptation leaves a pronounced imprint on the plant metabolome, even at a relatively small (i.e., within-population) spatial scale.

The metabolites that differed between home and away environments did not overlap with the metabolites that differed between acorn origins, indicating that the effect of local adaptation is distinct from the effect of acorn origin. The metabolites that differed between home and away environments included the amino acids alanine, glutamic acid, serine, glycine, threonine, and allothreonine. Alanine and glutamic acid are central to amino acid metabolism in plants (Forde and Lea 2007), and serine and glycine are integral to photorespiration and stomatal functioning in leaves (Less et al. 2011; Eisenhut et al. 2017). Threonine has been shown to be similar in function to allothreonine in plants (Muthuramalingam et al. 2018). Ethanolamine, which is a serine derivative and phospholipid precursor (Völz et al. 2021), and whose derivatives have been shown to play roles in plant-bacteria interactions (Coutinho et al. 2018) differed between home and away environments. Other metabolites that differed between home and away environments were the carbohydrates sucrose and scyllo-inositol, alpha-linolenic acid, quinic acid, beta-sitosterol, palmitic and malic acid. Alpha-linolenic acid is a precursor to plant oxylipins (Blée 2002; Wasternack and Feussner 2018). Quinic acid has been shown to accumulate in leaves under different stresses, although its exact function is unknown (Hijaz and Killiny 2019). Beta-sitosterol is a phytosterol and forms a component of plant cell membranes (Griebel and Zeier 2010). Palmitic acid is a lipid precursor (Napier and Graham 2010) and malic acid is a tricarboxylic acid pathway intermediate.

To help explore associations between plant growth and the metabolome or specific metabolites, we measured two plant traits—seedling height and leaf chlorophyll content. Yet, while we found disparate effects on the measured traits across microbiome treatments, no link could be made with the leaf metabolome results. For instance, the presence of increased tocopherol levels is linked to increased chlorophyll concentrations in the literature (Kanwischer et al. 2005). In our experiment, alpha-, beta- and gamma-tocopherols differed between canopy treatments. However, chlorophyll content did not differ between these treatments. Plants from acorn origins B and C generally overlapped in their leaf metabolomes but differed strongly in the two measured traits. Similarly, the leaf metabolome differed between the home and away treatments, but the two measured traits did not differ. This study utilized GC-MS, which is effective for measurements of primary metabolites (Lee et al. 2013). Assigning specific functions to primary metabolites is complicated as they are upstream of several derivatives with varied functions that are dynamic and depend on spatial and temporal factors (Gupta et al. 2022). To extend our findings, we hope that future studies will use advanced metabolomics techniques, such as LC-MS, to obtain information on downstream metabolites that can be concretely linked to plant health, stress conditions, and defence responses. While few if any other studies have tried to link plant metabolomes and plant traits within the context of microbiomes, several reviews have pointed out that fluctuations in the plant metabolome are not reflected linearly in plant functional traits, suggesting that the two methods provide different information on plant performance and fitness (Sardans et al. 2021; Walker et al. 2023). The plant metabolome can, for example, give more direct insights into ongoing plant physiological processes, detect changes not (yet) detectable as changes in plant traits, and provide a mechanistic understanding of why plant traits change (Díaz et al. 2016). Plant functional traits, in turn, are relevant for plant growth, ecological interactions and assessment of fitness outcomes. Thus, it might be fruitful to incorporate metabolomics approaches alongside functional trait analyses to obtain a holistic view of the interactions between plants and their microbiomes.

## Conclusion

 We found that acorn origin had a larger direct impact on the leaf metabolome than the soil and canopy environmental microbiomes, and that acorn origin and the environmental microbiome jointly shaped patterns of local adaptation. We hope that future studies will investigate the mechanisms behind microbiome-mediated local adaptation and look at how it affects plant metabolomes in natural ecosystems. These findings also have applied management implications, in terms of the use of local plant genotypes and plant breeding within the context of the local microbiome, with potential to sustainably improve forest health (Vivas et al. 2020). Likewise, the use of adapted microbiomes could be useful in afforestation and forest management practices (Baldrian et al. 2023; Addison et al. 2024). Utilizing metabolomics approaches alongside microbiome studies can also help disentangle the roles of specific microorganisms or specialized metabolites, which can help us design microbe-driven solutions in sustainable forestry management and agriculture.

## Supplementary Information

Below is the link to the electronic supplementary material.

Supplementary Material 1 (XLSX 66.8 KB)

Supplementary Material 2 (DOCX 120 KB)

## Data Availability

All data supporting this study are included in the manuscript and supplementary files.
