# Supplementary material for: The Effects of Acorn Origin, Environmental Microbiomes and Local Adaptation on the Leaf Metabolome
Source: J Chem Ecol. 2026 Feb 13;52(1):18. doi: 10.1007/s10886-026-01692-9 (PMC12904930; doi:10.1007/s10886-026-01692-9)

**Text S1.** Protocol for GC/MS analysis and subsequent data pre-processing

Gas chromatography system consisted of CTC Combi PAL Xt duo Autosampler (CTC Analytics AG, Switzerland), Agilent 7890A GC system (Agilent Technologies) and Pegasus HT time-of-flight mass spectrometer GC/TOFMS (Leco Corp., St Joseph, MI, USA). A split injection of 1 µL of derivatized sample was employed with 10:1 ratio. The liner used was a straight metal liner 5.2 × 6.3 × 78.5 mm packed with deactivated wool (Restek #21700) and the column used was 10 m × 0.18 mm, 0.18 μm, DB-5MS UI (J&W Scientific, Folsom, CA). The injector temperature was 270° C, and the helium flow rate was 1 mL/min. The column temperature was held at 70° C for 2 minutes, increased by 40° C/min to 320° C, and held for 2 minutes. The transfer line and the ion source temperatures were 250 ° C and 200 ° C, respectively. Detector voltage was 1450 V and ions were generated by a 70eV electron beam at an ionization current of 2.0 mA. Mass spectra were recorded at 30 scans per second with the range of m/z 50-800. Quality control samples were injected several times during the analysis and used to monitor instrument performance.

Mass spectra files were exported from the ChromaTOF software in NetCDF format to MATLAB R2020a (Mathworks, Natick, MA, USA), where all data pre-treatment procedures, such as base-line correction, chromatogram alignment, data compression and Multivariate Curve Resolution were performed. Deconvoluted peaks were annotated by comparisons of their retention index (RI) and mass spectra with libraries (Schauer et al 2005), based on SMC’s in-house library along with NIST library (National Institute of Standards and Technology, https://chemdata.nist.gov/), MoNa (MassBank of North America, <https://mona.fiehnlab.ucdavis.edu>), Golm metabolome database (http://gmd.mpimp-golm.mpg.de/) and MS-DIAL (http://prime.psc.riken.jp/compms/msdial/main.html).

**Table S1.** Overview of metabolites that significantly differed between soil and canopy microbiome treatments. Significance is indicated by asterisks besides the metabolite name, with *,**,*** indicating p-values < 0.05,0.01,0.001, respectively, for one-way ANOVA or Kruskal-Wallis tests.

| **Metabolites significantly differing between** | | | |
| --- | --- | --- | --- |
| **Soil microbiome treatments** | | **Canopy microbiome treatments** | |
| **Metabolites** | **Pairwise differences** | **Metabolites** | **Pairwise differences** |
| Allothreonine* | A-B | Ellagic acid* | C-S |
| Serine* | none | 1,5-anhydrosorbitol* | none |
|  | | Cycloartenol* | none |
|  |  | Glucuronic acid* | none |
|  |  | alpha-tocopherol* | none |
|  |  | delta-tocopherol* | none |
|  |  | gamma-tocopherol* | none |

**Table S2:** Metabolites of special interest that had a VIP>﻿ 1 in the OPLS-DA model with all three acorn origin classes. Significance of the among-group variance component is indicated by asterisks besides the metabolite name, with *,**,*** indicating p-values < 0.05,0.01,0.001, respectively, for one-way ANOVA or Kruskal-Wallis tests. If the overall test was significant, post-hoc pairwise comparisons were conducted using Tukey’s HSD or Dunn tests with Holm-Bonferroni correction for all three acorn combinations (A-B, A-C, and B-C), with adjusted p-values shown for each combination.

| **Metabolite** | **A-B** | **p-value (adj.)** | **A-C** | **p-value (adj.)** | **B-C** | **p-value (adj.)** |
| --- | --- | --- | --- | --- | --- | --- |
| 1,5-anhydrosorbitol |  |  |  |  |  |  |
| alpha-linolenic acid |  |  |  |  |  |  |
| beta-sitosterol |  |  |  |  |  |  |
| Citric acid* | Y | 0.0300 | Y | 0.0154 | N | 1 |
| delta-tocopherol*** | Y | 0.0030 | Y | 0.0020 | N | 0.9941 |
| DHAA* | N | 0.0563 | N | 0.148 | N | 0.8570 |
| Elaidic acid |  |  |  |  |  |  |
| Ellagic acid |  |  |  |  |  |  |
| Fructose |  |  |  |  |  |  |
| Glucose* | Y | 0.0187 | N | 0.3323 | N | 0.1962 |
| Glucuronic acid* | Y | 0.0186 | Y | 0.0027 | N | 0.8460 |
| Glyceric acid |  |  |  |  |  |  |
| Kaempferol* | N | 0.1081 | Y | 0.0134 | N | 0.5175 |
| myo-inositol |  |  |  |  |  |  |
| Oxoglutaric acid |  |  |  |  |  |  |
| Quinic acid |  |  |  |  |  |  |
| scyllo-inositol |  |  |  |  |  |  |
| Sedoheptulose-7-phosphate |  |  |  |  |  |  |
| Squalene |  |  |  |  |  |  |
| Succinic acid** | Y | 0.0310 | Y | 0.0037 | N | 0.5548 |
| Sucrose |  |  |  |  |  |  |
| UHMOGB…*** | Y | 0.0016 | Y | 0.0016 | N | 0.9948 |

**Table S3.** Metabolites of special interest that had a VIP>﻿ 1 in the OPLS-DA model with home and away samples. with all three acorn origin classes. Significance of the among-group variance component is indicated by asterisks besides the metabolite name, with *,**,*** indicating p-values < 0.05,0.01,0.001, respectively, for independent t-tests or Mann-Whitney U tests.

| **Metabolites** | **p-value** |
| --- | --- |
| Alanine*** | 0.0005 |
| Allothreonine** | 0.0022 |
| alpha-Linolenic acid* | 0.0379 |
| Aspartic acid | 0.1141 |
| beta-Sitosterol** | 0.0054 |
| Elaidic acid | 0.0588 |
| Ellagic acid | 0.1864 |
| Ethanolamine* | 0.0429 |
| Glutamic acid* | 0.6625 |
| Glyceric acid** | 0.0116 |
| Glycine** | 0.0016 |
| Malic acid* | 0.0057 |
| Palmitic acid* | 0.0102 |
| p-Coumaric acid | 0.0389 |
| Quinic acid* | 0.0945 |
| scyllo-inositol** | 0.0219 |
| Serine* | 0.0025 |
| Sucrose** | 0.0126 |
| Threonine** | 0.0038 |

**Table S4.** A summary table of plant height and leaf chlorophyll content. Shown are means of plant traits in the different treatments, as well as the p-value of the ANOVA testing for differences between treatment levels

| Treatment | Plant height (cm) | ANOVA P-value | Leaf chlorophyll content (cci) | ANOVA P-value |
| --- | --- | --- | --- | --- |
| Acorn origin A | 13.76 | <0.01 | 18.45 | <0.01 |
| Acorn origin B | 14.80 |  | 21.48 |  |
| Acorn origin C | 17.26 |  | 17.62 |  |
| Soil microbiome A | 14.67 | 0.094 | 18.85 | <0.01 |
| Soil microbiome B | 16.52 |  | 19.31 |  |
| Soil microbiome C | 14.29 |  | 17.71 |  |
| Soil microbiome control | 15.63 |  | 20.85 |  |
| Canopy microbiome A | 15.01 | 0.99 | 18.60 | 0.80 |
| Canopy microbiome B | 15.08 |  | 19.63 |  |
| Canopy microbiome C | 15.29 |  | 18.77 |  |
| Canopy microbiome control | 15.34 |  | 19.64 |  |
| Home environment | 15.34 | 0.84 | 20.54 | 0.36 |
| Away environment | 16.00 |  | 18.63 |  |

**Table S5.** The results of pairwise analyses performed on traits that differed significantly in the ANOVA across different treatments. “*” besides their name indicates significance.

| **Treatment** | **Combination** | **Variable** |
| --- | --- | --- |
| Soil | AB | Chlorophyll Content* |
| Soil | AC | Chlorophyll Content |
| Soil | AS | Chlorophyll Content* |
| Soil | BC | Chlorophyll Content* |
| Soil | BS | Chlorophyll Content |
| Soil | CS | Chlorophyll Content* |
| Acorn | AB | Height |
| Acorn | AC | Height* |
| Acorn | BC | Height* |
| Acorn | AB | Chlorophyll Content* |
| Acorn | AC | Chlorophyll Content |
| Acorn | BC | Chlorophyll Content* |

**Figure S1.** Changes in the leaf metabolome of oak seedlings in plants grown from different acorn origins visualized in an OPLS-DA score plot. The OPLS-DA model was fit to two components (1+1+0, one predictive and one orthogonal), R^2^x(cum) = 0.148 and Q^2^cum = 0.304.


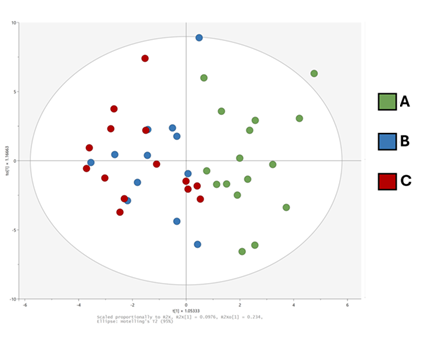


**Figure S2.** Changes in the leaf metabolome of oak seedlings in plants grown from different acorn origins visualized in an OPLS-DA score plot (1) without acorn origins specified and (2) with acorn origins specified beside each sample. The OPLS-DA model was run only with two components (one predictive and one orthogonal), R^2^X(cum) = 0.33, Q^2^(cum) = 0.109


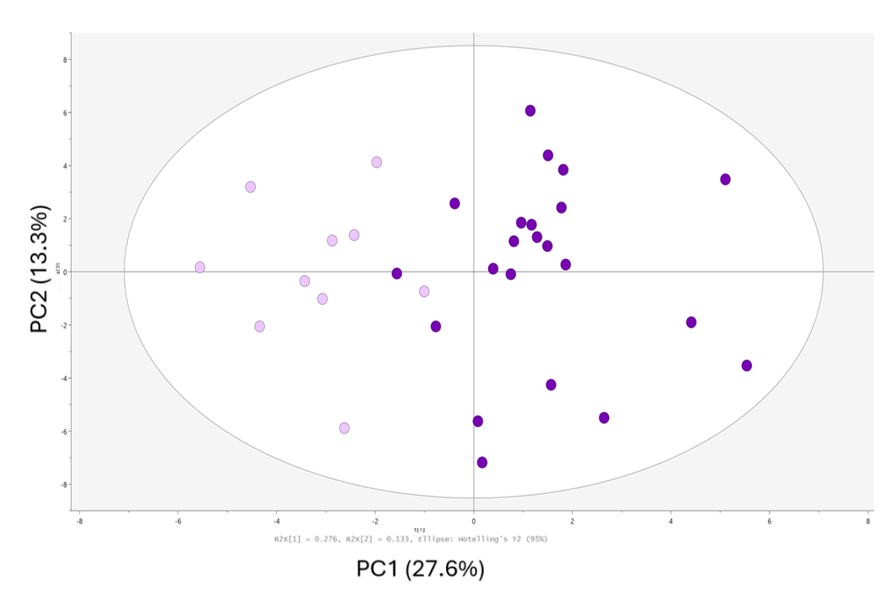


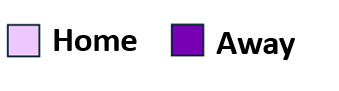

Supplement: Supplementary file 2 — Supplementary Material 2 (DOCX 120 KB) [file 10886_2026_1692_MOESM2_ESM.docx]
